# Understanding and Quantifying the Benefit of Graded Aluminum Gallium Nitride Channel High-Electron Mobility Transistors

**DOI:** 10.3390/mi15111356

**Published:** 2024-11-08

**Authors:** François Grandpierron, Elodie Carneiro, Lyes Ben-Hammou, Jeong-Sun Moon, Farid Medjdoub

**Affiliations:** 1 Institute of Electronics, Microelectronics and Nanotechnology, IEMN, 59652 Villeneuve-d’Ascq, France; elodie.carneiro@univ-lille.fr (E.C.); lyes.benhammou@univ-lille.fr (L.B.-H.); farid.medjdoub@univ-lille.fr (F.M.); 2 HRL Laboratories, Malibu, CA 90265, USA; jmoon@hrl.com

**Keywords:** AlGaN graded channel, HEMT, transconductance, linearity, simulations

## Abstract

Graded AlGaN channel High-Electron Mobility Transistor (HEMT) technology is emerging as a strong candidate for millimeter-wave applications, as superior efficiency and linearity performances can be achieved. In this paper, graded channel AlGaN/GaN HEMTs are investigated with the aim of further understanding the benefit of the graded AlGaN channel compared to more conventional GaN channel HEMTs. Our study employed a comprehensive simulation workflow including an extensive calibration of direct current (DC), S-parameter, large signal, and linearity characteristics at 30 GHz. Through device modeling and implementation of circuit-level simulation using Advanced Design System (ADS, 2023) software, both linearity and large signal performances could be mimicked remarkably. In agreement with previous studies, the results show that graded channel technology allows for a modified electron confinement leading to a 3D electron gas (3DEG). Consequently, the electric field peak inside of the channel is reduced without degrading the radio frequency (RF) performance, as the electron velocity is improved, thus offering a more linear transconductance and better linearity performances. As a result, for graded AlGaN channel HEMTs, a 6 dB output power back-off from peak power-added efficiency (PAE) is needed to achieve a carrier with a third-order intermodulation (C/IM3) ratio of 30 dBc against 9 dB for conventional AlGaN/GaN HEMTs with a lower associated PAE.

## 1. Introduction

Gallium Nitride (GaN) High-Electron Mobility Transistors (HEMTs) have drawn great attention owing to their potential for high-power millimeter-wave (mmW) applications. To respond to this trend, a number of GaN HEMT technologies have made great strides and shown significant progress to achieve high device efficiency. However, combining high linearity and efficiency in high-power GaN devices is still challenging in the mm-wave range. For instance, ultrathin AlN barrier GaN HEMTs exhibiting outstanding Ka-band performance [1,2], such as continuous wave (CW) power-added efficiency (PAE) above 65% under output power (P_OUT_) > 3 W/mm at a drain-source voltage (V_DS_) of 20 V [2]. N-polar GaN devices showed excellent performances at 94 GHz with a record PAE of 53.4% and a P_OUT_ of 3.4 W/mm at V_DS_ = 12 V [3,4]. Linearity measurements at 30 GHz also demonstrate the capabilities of N-polar technology, achieving a C/I ratio above 37 dBc but under 10 dB output power back-off conditions, which results in a severely degraded PAE of around 15% [5,6]. FinFET or BRIDGE-FET technologies are another emerging approach to achieving linear devices. Results indicate that optimizing gate-metal design offers the possibility to boost the linearity, resulting in a flat transconductance [7,8,9,10]. However, it can be pointed out that adding metals introduces additional capacitances, which in turn may degrade the overall RF performances, and they can be complex to implement on an industrial scale.

In recent years, graded channel (GC) AlGaN/GaN transistors have shown state-of-the-art results up to the W-band [11] while also exhibiting excellent linearity characteristics at 30 GHz [12]. The structure incorporates a 60 nm T-gate with a mini-field plate to reduce the peak electric field with regrown n+ ohmic contacts to maximize the power gain and the drain current density [13]. The epi-structure consists of a standard AlGaN barrier layer of 15 nm, a 20 nm thick GaN channel, and a 1-micron AlGaN back barrier to confine electrons in the two-dimensional electron gas (2DEG) and prevent short-channel effects with short gate lengths. A key feature of this technology is the insertion of a thin, graded AlGaN layer with an Al composition ranging from 0% to 10% between the AlGaN barrier and the GaN channel. It is worth noting that the graded channel architecture is fully reproducible, with no reported issues related to additional processing steps. This design enables high linear transconductance exceeding 500 mS/mm, with F_T_/F_MAX_ values of 150 GHz and 300 GHz, respectively [13]. The graded AlGaN channel represents the best combination of high efficiency and linearity to date for Ga-polar HEMTs, achieving a C/I ratio above 30 dBc while maintaining a high PAE of 50% [12]. To further understand the benefit of this technology, several studies have been carried out showing that the graded AlGaN channel induces a distributed three-dimensional charge (3DEG) profile and eliminates the abrupt bandgap compared to a conventional AlGaN/GaN heterojunction [14,15,16,17]. The formation and distribution of charges along the graded channel involve multiple mechanisms, including the reduction of the electric field in the channel at the gate edge towards the drain without additional induced capacitances. Moreover, G_M_ shows a much flatter shape beyond the peak compared to conventional AlGaN/GaN HEMTs, where the G_M_ exhibits a sharp peak followed by an abrupt fall-off, leading to poorer linearity [18,19]. This phenomenon is mainly due to the reduced electron velocity from hot phonon scattering effects at high carrier density [20,21] and an increase in source access resistance [22]. The capacitances are also affected, and they are lower compared to those in AlGaN/GaN HEMTs. Notably, the gate-source capacitance (C_GS_) exhibits a softer, less abrupt characteristic, which can improve device linearity [18,23].

In this paper, we introduce a simulation method that spans from the device to the circuit level, which enables mimicking the loadpull and linearity characteristics for a better understanding of the device technology. Subsequently, a proper comparison of graded AlGaN channel HEMTs and conventional AlGaN/GaN HEMTs is carried out, reflecting the main benefits through large signal and linearity characteristics.

## 2. Workflow Validation and Data Calibration

**Figure 1:**
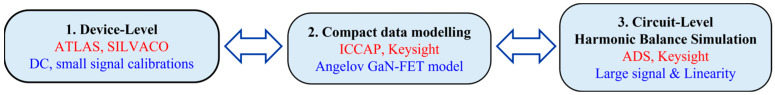
Flowchart showing three different levels involved in the simulation workflow.

The simulation workflow involves three levels: device-level simulation (level 1), compact data modeling (level 2), and circuit-level simulation (level 3), as shown in the flowchart below (Figure 1). It is important to note that all three levels of this workflow are interconnected, allowing data to be retrofitted at each level.

Level 1 is a device-level Technology Computer-Aided Design (TCAD) simulation performed using SILVACO ATLAS (2024, version 5.38.0.R). It involves the device design structure, especially the epitaxial layer compositions and thicknesses, followed by transistor properties, such as 2DEG properties, electron mobility (µ), and carrier concentration (n_s_) via physics-based models. However, calibration of DC and RF characteristics against the experimental results is necessary to validate the simulated device. Shockley–Read–Hall recombination (SRH), Fermi–Dirac (FERMI) statistics, the parallel field mobility (FLDMOB) model, and the temperature-dependent low field model (Farahmand Modified Caughey Thomas—FMCT) are used to achieve device calibration. To complete level 1, TCAD simulation has been performed on HRL devices [12,13]. The active regions consist of a 1 μm Al_0.04_Ga_0.96_N buffer, a 20 nm thick GaN channel layer, and a 6 nm thick linearly graded AlGaN channel layer with an Al composition spanning from 0% to 10%. The barrier layer is 15 nm thick Al_0.25_Ga_0.75_N with n^+^ regrown GaN regions to achieve low contact resistances. The T-gate length is 50 nm, and the source-drain contact distance is 0.5 µm. The carrier concentration is 9.0 × 10^12^ cm^−2^, and the mobility is 1500 cm^2^/Vs.

**Figure 2:**
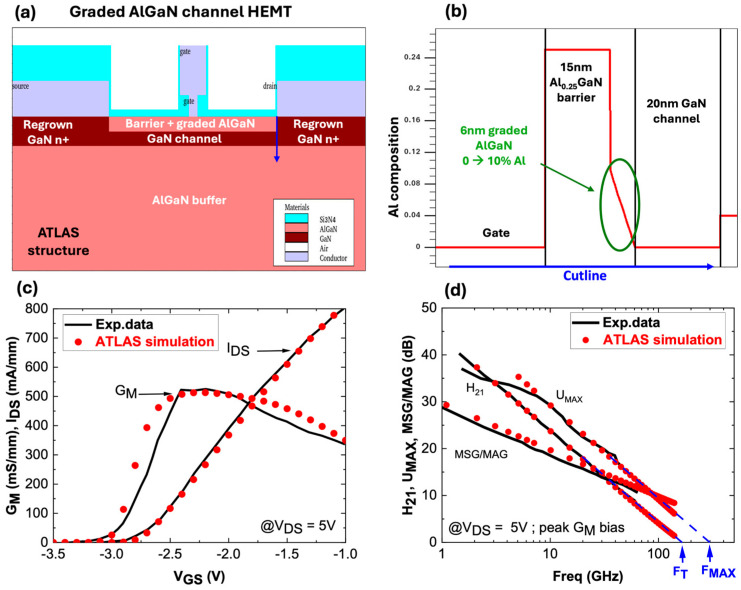
(**a**) Simulated graded AlGaN channel, (**b**) Al-profiles along the structure, (**c**) simulated DC, and (**d**) RF small signal characteristics vs. exp. data [12,13].

**Figure 3:**
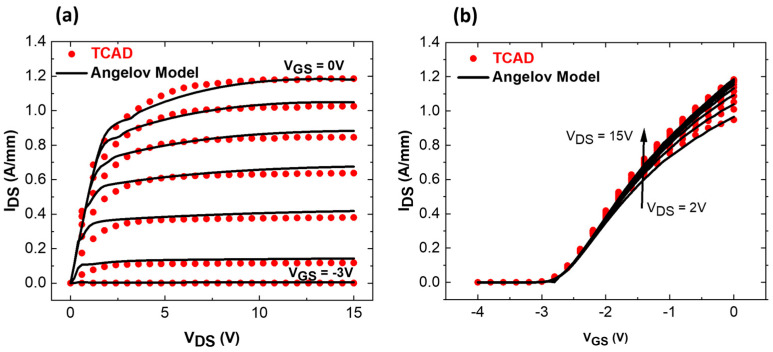
DC fitting vs. Angelov model (**a**) I_D_V_D_, (**b**) I_D_V_G_ for graded AlGaN channel HEMT.

Figure 2a presents the simulated structure in ATLAS. A cutline is used through the structure under the gate to the GaN channel, showing the Al-profiles of each layer (Figure 2b). Corresponding DC and RF small signal calibrations with experimental data are shown in Figure 2c,d. Excellent matching between the experimental data and TCAD simulations could be obtained. A similar transconductance shape (G_M_ peak = 515 mS/mm) and an I_D_V_G_ curve with an identical maximum current have been reproduced, resulting in F_T_ (165 GHz TCAD vs. 158 GHz exp. data) and F_MAX_ (290 GHz TCAD vs. 308 GHz exp. data) fully in agreement with the experimental data. Level 2 involves the generation of a compact and non-linear transistor model for TCAD-simulated data. The extraction of compact model parameters is completed using a commercially available software ICCAP (2023). We selected the Angelov GaN Model, a well-established mathematical model dedicated to GaN FETs [24,25]. Parameter extraction includes several steps and requires multiple iterations to reach sufficient matching between TCAD data and the model [26]. Figure 3 shows the qualitative DC fitting of TCAD data with the Angelov model imported into ICCAP. After satisfactory fitting, the model parameters are exported as “*.mps*” files, which are readable by the circuit-level simulator ADS.

**Figure 4:**
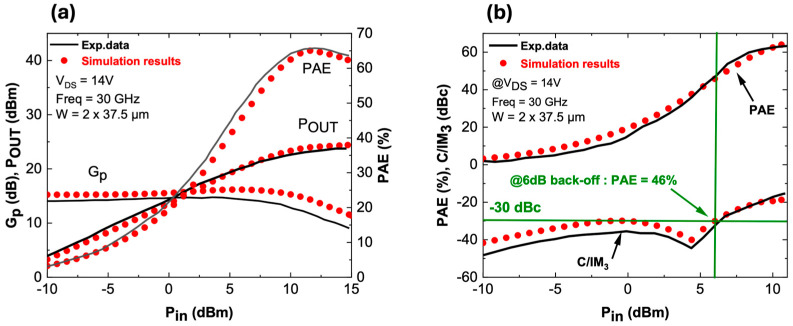
(**a**) One-tone large signal simulation vs. exp. data, (**b**) two-tone linearity simulation vs. exp. data [12].

Level 3 is a circuit-level simulation performed using ADS. The compact, non-linear model from ICCAP to ADS is a custom-made GaN HEMT, which acts as the device under test (DUT) for harmonic balance simulations to predict active loadpull and two-tone linearity characteristics at 30 GHz. Figure 4 shows a strong agreement between simulated large signal and linearity characteristics with the experimental data [12]. It is worth noting that extensive parameter tuning—over hundreds of iterations—was required to achieve a good match between TCAD data and the Angelov model. In particular, the capacitances C_GS_, C_GD_, and C_DS_ played a key role in achieving these results. Furthermore, the Angelov model exported to ADS is calibrated only for a specific bias point (in this case, V_DS_ = 14 V); operation at different voltages would require further parameter adjustment.

A comparable max PAE of 65% at V_DS_ = 14 V is obtained (Figure 4a) with an associated P_OUT_ of 3.3 W/mm (3.0 W/mm reported) with a quasi-identical impedance matching of I = 0.763 > 23° (0.76 > 28° reported). Similarly, a close matching of C/I ratio is observed, which is found to be greater than 30 dBc with an associated PAE of 46% at 6 dB output power back-off from the peak PAE, as shown in Figure 4b.

## 3. Graded Channel vs. AlGaN/GaN HEMT

**Figure 5:**
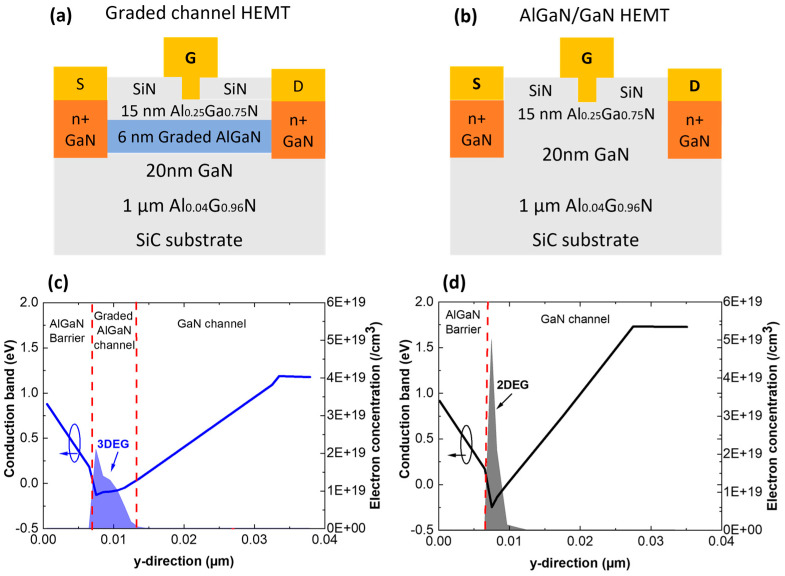
(**a**) Simulated graded AlGaN channel HEMT, (**b**) conventional AlGaN/GaN HEMT, (**c**) extracted conduction band diagram of graded AlGaN channel, (**d**) conventional AlGaN/GaN HEMT.

Based on this extensive calibration, including DC, RF, loadpull, and linearity performances, a comparison of the graded AlGaN channel with a conventional AlGaN/GaN HEMT has been carried out. The full workflow has been applied in similar structures with and without the graded AlGaN channel. Figure 5a,b illustrate the two structures designed in TCAD, along with their corresponding conduction band diagram (eV) and electron concentration (/cm^3^), as shown in Figure 5c,d. These data were obtained directly from cutlines through both structures with no applied bias. The conduction band diagram for the graded AlGaN channel (Figure 5c) reveals a broader electron distribution compared to the conventional AlGaN/GaN HEMT (Figure 5d). In the standard AlGaN/GaN HEMT, a typical carrier distribution is observed at the heterojunction, where electrons are tightly confined at the interface, resulting in a sharp electron density between the AlGaN barrier and the GaN channel. In contrast, as reported [17,27], the graded structure confines electrons in a volume charge density, which locally reduces the charge density in the channel. This effect causes electron spreading across the graded channel, forming a 3D electron gas (3DEG) over an approximate width of 6 nm. Thus, a higher 2DEG of 1.5 × 10^13^ cm^−2^ was fixed for the conventional AlGaN/GaN HEMT with a mobility of 1500 cm^2^/Vs.

**Figure 6:**
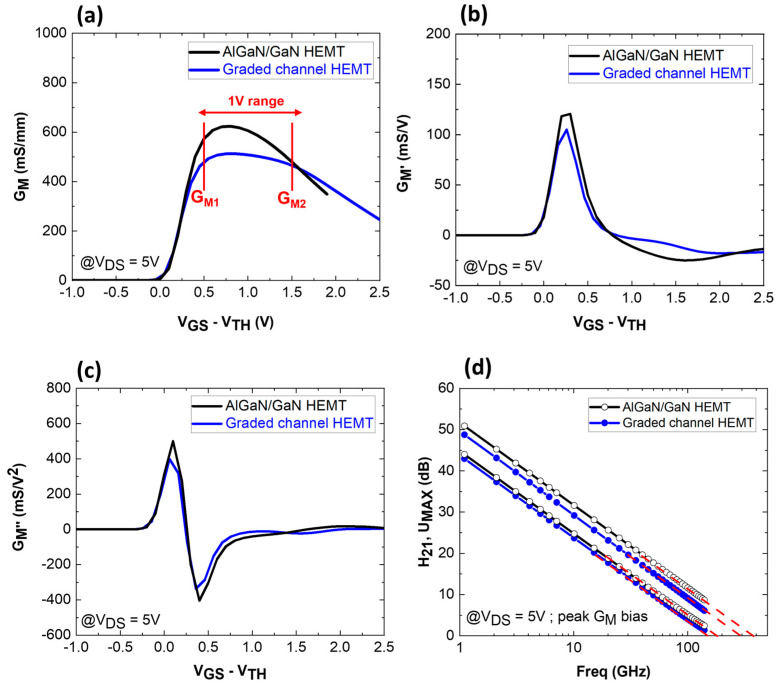
Simulated G_M_ (**a**), G_M′_ (**b**), G_M″_ (**c**) of graded channel and AlGaN/GaN HEMTs and (**d**) RF small signal characteristics comparison.

Figure 6 presents a comparison of G_M_ (Figure 6a), its first derivative (G_M′_) (Figure 6b), its second derivative (G_M″_) (Figure 6c), and small signal characteristics (Figure 6d) at V_DS_ = 5 V for both structures. The plots have been aligned to compensate for the shift in the threshold voltage that results from the different 2DEG properties. Higher transconductance as well as F_T_/F_MAX_ are found for the AlGaN/GaN HEMT. This is due to the higher carrier concentration in the channel. However, the graded channel enables a much flatter G_M_, indicating better linearity performance. From Figure 6a, a simple calculation of the difference in the G_M peak_ and the G_M_ at a voltage range of 1 V can be applied for both structures. This parameter can define a “roll-off” factor. For the graded AlGaN channel, the difference is equal to 93 mS/mm (18% decrease), whereas for the AlGaN/GaN HEMT, the difference is 245 mS/mm (39% decrease). This confirms the better flatness of G_M_ for the graded channel HEMT. The improved linearity is also reflected on G_M′_ and G_M″_. In fact, the amplitudes of the derivatives are smaller for the graded channel, suggesting less of an impact of intermodulation distortions.

**Figure 7:**
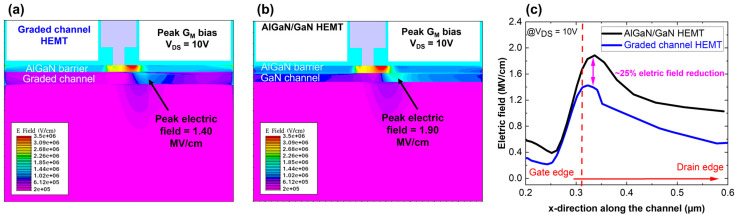
Electric field distribution for graded channel (**a**), AlGaN/GaN HEMT (**b**) and electric field profiles comparison (**c**), extracted from ATLAS.

To further study the formation of the 3DEG and its impact on the G_M_ shape, the lateral electric field has been extracted for both structures at peak G_M_ bias and V_DS_ = 10 V along the channel (Figure 7). From TCAD simulations, it appears that the graded AlGaN channel HEMT (Figure 7a) enables a reduction of the electric field compared with the standard GaN channel (Figure 7b). The peak electric field in the channel is equal to 1.40 MV/cm against 1.90 MV/cm for the AlGaN/GaN HEMT. Electric field profiles have been extracted using a cutline along the channel from the gate to the drain edge. Figure 7c shows that the graded channel allows for a 25% reduction in the peak field, which is consistent with previous reports [27,28].

**Figure 8:**
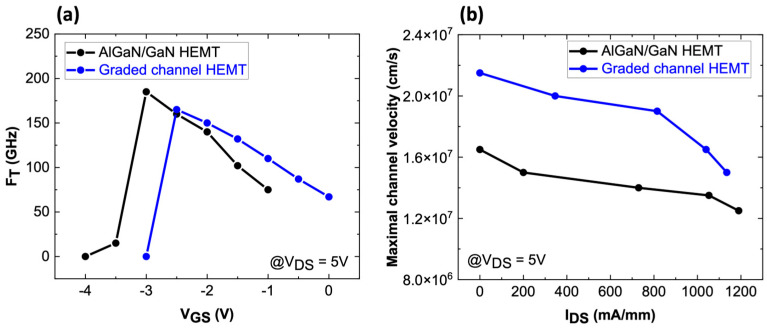
(**a**) Simulated F_T_ comparison between AlGaN/GaN and graded channel HEMT at V_DS_ = 5 V for different V_GS_, (**b**) simulated maximal channel velocity at different current levels.

In terms of RF small signal performances, the cut-off frequency (F_T_) was also extracted from S-parameter simulations for various V_GS_ for both devices. As shown in Figure 6, the graded channel has a more linear transconductance, enabling it to maintain high F_T_ values over a wider V_GS_ range (Figure 8a). In contrast, the AlGaN/GaN device achieves a higher F_T_ peak but over a narrower V_GS_ range. This behavior is attributed to the ability of the graded channel to handle greater electron velocity saturation compared with the conventional AlGaN/GaN structure. Specifically, the channel velocity has been extracted from TCAD simulations for both structures.

**Figure 9:**
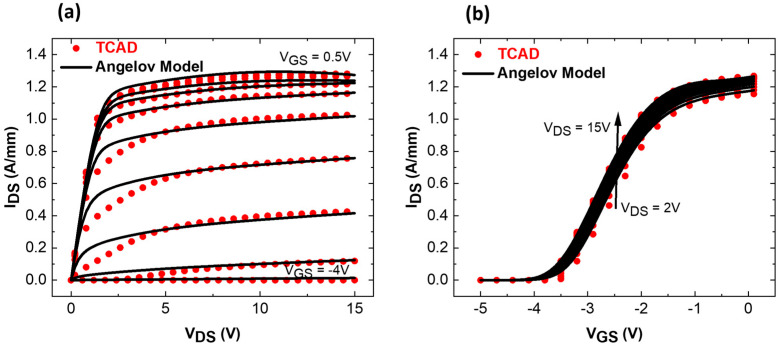
DC fitting vs. Angelov model (**a**) I_D_V_D_, (**b**) I_D_V_G_ for AlGaN/GaN HEMT.

Figure 8b indicates that the graded channel is less affected by the saturation velocity limitation caused by hot phonon scattering. This is due to the 3DEG configuration, resulting in a more linear G_M_ response. To quantify the benefits of the graded AlGaN channel, the simulation workflow described initially was applied to the simulated AlGaN/GaN HEMT. Figure 9 presents the DC fitting between TCAD data and the Angelov model (ICCAP, level 2). The model was computed and exported to ADS to compare the large signal and linearity characteristics of both devices.

**Figure 10:**
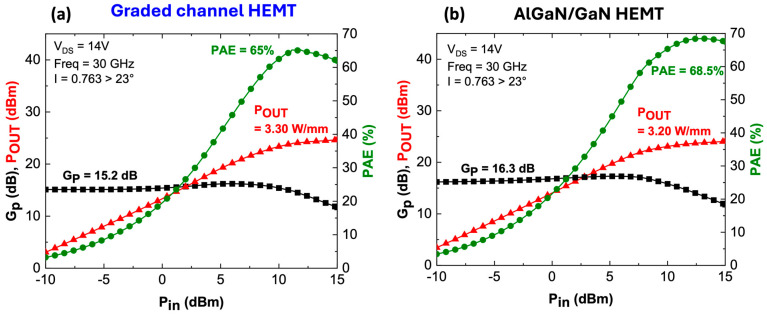
Large signal performance comparison between graded channel (**a**) and AlGaN/GaN HEMT (**b**).

**Figure 11:**
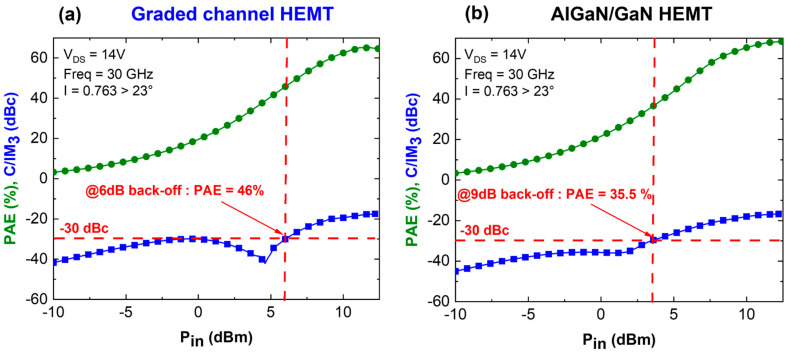
Linearity performance comparison between graded channel (**a**) and AlGaN/GaN HEMT (**b**).

Figure 10 depicts the large signal comparison between both devices at 30 GHz using an identical impedance matching. At the same bias (V_DS_ = 14 V), a higher PAE (68.5%) is observed for the conventional AlGaN/GaN HEMTs with a comparable P_OUT_. This is consistent with the higher power gain for the conventional GaN channel (16.3 dB vs. 15.2 dB for the graded channel) owing to a higher peak transconductance. On the other hand, the linearity performance (Figure 11) at P_IN_ = 12 dBm reveals that the graded AlGaN channel has a C/I ratio of 17.5 dBc near the maximum PAE.

This is higher than the conventional AlGaN/GaN HEMT, which delivers a C/I ratio of 16.5 dBc, showing a degraded linearity near the maximum PAE. When focusing on the linear regime, at C/I = 30 dBc, a 6 dB output power back-off is required for the graded AlGaN channel HEMTs delivering an associated PAE of 46%. In contrast, conventional AlGaN/GaN HEMTs reach a C/I = 30 dBc at 9 dB output power back-off with an associated PAE of 35.5%. A significantly higher back-off is needed from the maximum PAE to achieve a linear regime for conventional GaN channel HEMTs, which is attributed to the higher electric field peak at the gate edge. Indeed, spreading the electric field enables mitigating hot electron effects, as energetic carriers can interact non-linearly with the lattice, leading to phenomena like impact ionization and trap generation, which introduce distortion. Thus, the graded AlGaN channel HEMT offers an advantageous performance compared to standard AlGaN/GaN HEMTs, enabling it to combine a high PAE with excellent linearity, as required for future mmW applications.

## 4. Conclusions

For a high frequency beyond 30 GHz, a graded AlGaN channel HEMT is an attractive solution to overcome the trade-off between high linearity and high power-added efficiency compared to the conventional AlGaN/GaN HEMT. In this work, large signal and linearity performances of promising graded channel-based HEMTs fabricated using HRL have been successfully reproduced by employing a hybrid simulation method for the first time. The simulation approach consists of three different levels, including ATLAS (SILVACO), compact transistor modeling (Angelov GaN Model, ICCAP), and circuit-level simulation (ADS). Subsequently, a comparison with conventional AlGaN/GaN HEMTs shows the benefit of the graded AlGaN channel HEMTs. Indeed, the formation of a 3DEG enables spreading the electric field (similarly to field plates but without the drawback of a parasitic capacitance penalty) and improving the channel velocity, thus enabling flat G_M_ and the device’s linearity. The linearity and power-added efficiency combination improvement could be quantified. The proposed method allows for further understanding of advanced RF devices in operational conditions. This may guide engineers in optimizing device architecture for practical applications and lead to more sustainable manufacturing practices.

## Data Availability

The original contributions presented in the study are included in the article, further inquiries can be directed to the corresponding author.

## References

[1] Harrouche K., Kabouche R., Okada E., Medjdoub F. (2019). High Performance and Highly Robust AlN/GaN HEMTs for Millimeter-Wave Operation. IEEE J. Electron Devices Soc..

[2] Harrouche K., Venkatachalam S., Ben-Hammou L., Grandpierron F., Okada E., Medjdoub F. (2023). Low Trapping Effects and High Electron Confinement in Short AlN/GaN-On-SiC HEMTs by Means of a Thin AlGaN Back Barrier. Micromachines.

[3] Romanczyk B., Wienecke S., Guidry M., Li H., Hestroffer K., Ahmadi E., Zheng X., Keller S., Mishra U.K. Mm-Wave N-Polar GaN MISHEMT with a Self-Aligned Recessed Gate Exhibiting Record 4.2 W/mm at 94 GHz on Sapphire. Proceedings of the 2016 74th Annual Device Research Conference (DRC).

[4] Collins H., Akso E., Clymore C.J., Khan K., Hamwey R., Hatui N., Guidry M., Keller S., Mishra U.K. (2024). N-Polar Deep Recess GaN HEMT with a TiN Schottky Gate Contact Demonstrating 53.4% PAE and 3.7 W/mm Associated P_out_ at 94 GHz. IEEE Microw. Wirel. Technol. Lett..

[5] Romanczyk B., Guidry M., Zheng X., Shrestha P., Li H., Ahmadi E., Keller S., Mishra U.K. (2021). Evaluation of Linearity at 30 GHz for N-Polar GaN Deep Recess Transistors with 10.3 W/mm of Output Power and 47.4% PAE. Appl. Phys. Lett..

[6] Guidry M., Romanczyk B., Li H., Ahmadi E., Wienecke S., Zheng X., Keller S., Mishra U.K. Demonstration of 30 GHz OIP3/PDC > 10 dB by Mm-Wave N-Polar Deep Recess MISHEMTs. Proceedings of the 2019 14th European Microwave Integrated Circuits Conference (EuMIC).

[7] Shinohara K., King C., Regan D., Regan E., Carter A., Arias A., Bergman J., Urteaga M., Brar B., Cao Y. Tu4E-4 Multi-Channel Schottky-Gate BRIDGE HEMT Technology for Millimeter-Wave Power Amplifier Applications. Proceedings of the 2022 IEEE/MTT-S International Microwave Symposium—IMS 2022.

[8] Shinohara K., King C., Carter A.D., Regan E.J., Arias A., Bergman J., Urteaga M., Brar B. (2018). GaN-Based Field-Effect Transistors with Laterally Gated Two-Dimensional Electron Gas. IEEE Electron Device Lett..

[9] Choi W., Chen R., Levy C., Tanaka A., Liu R., Balasubramanian V., Asbeck P.M., Dayeh S.A. (2020). Intrinsically Linear Transistor for Millimeter-Wave Low Noise Amplifiers. Nano Lett..

[10] Shinohara K., King C., Regan E., Gomez M.P., Bergman J., Carter A., Arias A., Urteaga M., Brar B., Page R. (2019). (Invited) GaN-Based Multiple 2DEG Channel BRIDGE (Buried Dual Gate) HEMT Technology for High Power and Linearity. ECS Trans..

[11] Moon J.-S., Grabar B., Wong J., Dao C., Arkun E., Tai H., Fanning D., Miller N.C., Elliott M., Gilbert R. (2023). *W*-Band Graded-Channel GaN HEMTs with Record 45% Power-Added-Efficiency at 94 GHz. IEEE Microw. Wirel. Technol. Lett..

[12] Moon J., Wong J., Grabar B., Antcliffe M., Chen P., Arkun E., Khalaf I., Corrion A., Post T. Novel High-Speed Linear GaN Technology with High Efficiency. Proceedings of the 2019 IEEE MTT-S International Microwave Symposium (IMS).

[13] Moon J.-S., Grabar B., Wong J., Chuong D., Arkun E., Morales D.V., Chen P., Malek C., Fanning D., Venkatesan N. (2021). Power Scaling of Graded-Channel GaN HEMTs with Mini-Field-Plate T-Gate and 156 GHz f_T_. IEEE Electron Device Lett..

[14] Bajaj S., Yang Z., Akyol F., Park P.S., Zhang Y., Price A.L., Krishnamoorthy S., Meyer D.J., Rajan S. (2017). Graded AlGaN Channel Transistors for Improved Current and Power Gain Linearity. IEEE Trans. Electron Devices.

[15] Rajan S., DenBaars S.P., Mishra U.K., Xing H.G., Jena D. (2006). Electron Mobility in Graded AlGaN Alloys. Appl. Phys. Lett..

[16] Sohel S.H., Bajaj S., Razzak T., Meyer D.J., Rajan S. Design of Graded AlGaN Channel Transistors for Improved Large-Signal Linearity. Proceedings of the International Conference on Compound Semiconductor Manufacturing Technology.

[17] Sung Park P., Nath D.N., Krishnamoorthy S., Rajan S. (2012). Electron Gas Dimensionality Engineering in AlGaN/GaN High Electron Mobility Transistors Using Polarization. Appl. Phys. Lett..

[18] Venkatesan N., Silva-Oelker G., Fay P. Graded-Channel GaN-Based HEMTs for High Linearity Amplifiers at Millimeter-Wave. Proceedings of the 2019 IEEE BiCMOS and Compound Semiconductor Integrated Circuits and Technology Symposium (BCICTS).

[19] Tarakji A., Fatima H., Hu X., Zhang J.-P., Simin G., Khan M.A., Shur M.S., Gaska R. (2003). Large-Signal Linearity in III-N MOSDHFETs. IEEE Electron Device Lett..

[20] Oxley C.H., Uren M.J., Coates A., Hayes D.G. (2006). On the Temperature and Carrier Density Dependence of Electron Saturation Velocity in an AlGaN/GaN HEMT. IEEE Trans. Electron Devices.

[21] Khurgin J.B., Bajaj S., Rajan S. (2015). Elastic Scattering by Hot Electrons and Apparent Lifetime of Longitudinal Optical Phonons in Gallium Nitride. Appl. Phys. Lett..

[22] Palacios T., Rajan S., Chakraborty A., Heikman S., Keller S., DenBaars S.P., Mishra U.K. (2005). Influence of the Dynamic Access Resistance in the g_m_ and f_T_ Linearity of AlGaN/GaN HEMTs. IEEE Trans. Electron Devices.

[23] Moon J.-S., Wong J., Grabar B., Antcliffe M., Chen P., Arkun E., Khalaf I., Corrion A., Chappell J., Venkatesan N. (2020). 360 GHz f_MAX_ Graded-Channel AlGaN/GaN HEMTs for mmW Low-Noise Applications. IEEE Electron Device Lett..

[24] Angelov I., Andersson K., Schreurs D., Xiao D., Rorsman N., Desmaris V., Sudow M., Zirath H. Large-Signal Modelling and Comparison of AlGaN/GaN HEMTs and SiC MESFETs. Proceedings of the 2006 Asia-Pacific Microwave Conference.

[25] Angelov I., Grabinski W., Nauwelaers B., Schreurs D. (2006). Empirical FET Models. Transistor Level Modeling for Analog/RF IC Design.

[26] Avolio G., Vadalà V., Angelov I., Raffo A., Marchetti M., Vannini G., Schreurs D. (2017). A Procedure for the Extraction of a Nonlinear Microwave GaN FET Model. Int. J. Numer. Model..

[27] Venkatesan N., Moon J.-S., Fay P. Electric Field Engineering in Graded-Channel GaN-Based HEMTs. Proceedings of the 2021 IEEE BiCMOS and Compound Semiconductor Integrated Circuits and Technology Symposium (BCICTS).

[28] Fay P., Moon J.-S., Rajan S. (2022). III-N Polarization-Graded Transistors for Millimeter-Wave Applications—Understanding and Future Potential. Appl. Phys. Lett..

